# Diabetes trends in youth

**DOI:** 10.1111/1753-0407.13382

**Published:** 2023-03-30

**Authors:** Zachary Bloomgarden, Robert Rapaport

**Affiliations:** ^1^ Department of Medicine, Division of Endocrinology, Diabetes and Bone Disease Icahn School of Medicine at Mount Sinai New York New York USA; ^2^ Division of Pediatric Endocrinology and Diabetes Icahn School of Medicine at Mount Sinai New York New York USA; ^3^ Division of Pediatric Endocrinology and Diabetes Kravis Children's Hospital at Mount Sinai New York New York USA

Type 2 diabetes (T2D) prevalence in the 10‐ to 19‐year‐old population has doubled over the past two decades in the United States.^1^ It is present in nearly 2 persons per 1000 among Black and American Indian youth, in about 1 per 1000 among Hispanic youth, but in 0.2 per 1000 among non‐Hispanic white youth, the incidence of T2D has similarly doubled among adolescents in the United States over this period, from 9 to 18 cases per 100 000 per year, with incidence again particularly high in Black and American Indian youth.^2^

The incidence of type 1 diabetes (T1D) in the United States also increased, from 20 to 22 per 100 000 persons age <20 during the same period,^2^ and its prevalence increased from 1.5 to 2.2 per 1000,^1^ with a different racial and ethnic composition, more common in non‐Hispanic white, then Black, then Hispanic, then Asian and then American Indian.

The numbers of youth with T1D and T2D in 2017 were 185 000 and 28 000, respectively, and, based on these increasing prevalence and incidence rates, the numbers are projected to be 335 000 youth with T1D and 220 000 with T2D in 2060, increases of 65% and 673%, respectively.^3^

Similarly, T2D and T1D in youth are increasing in incidence and prevalence worldwide. The number of youths with T2D is more than twice as great in China and 1.4‐fold higher in India than in the United States based on the larger population of these countries, but with the United States showing the highest reported prevalence in a recent worldwide survey (Table 1).^4^

**TABLE 1:** Worldwide T2D Prevalence, from Data in Ref. 4

Country	Cases	Person‐years	Cases/100000 person‐years
United States	355	2 575 000	13.8
Hong Kong	104	1 188 800	8.7
Taiwan	186	2 862 983	6.5
India	92	1 818 413	5.1
Hungary	92	1 818 413	5.1
China	105	2 900 552	3.6
Kuwait	32	1 252 434	2.6
New Zealand	52	2 888 888	1.8
Canada	227	14 717 870	1.5
Australia	135	10 384 615	1.3
Russia	272	27 200 000	1.0
United Kingdom	94	13 008 432	0.7
Austria	8	1 263 740	0.6
Finland	8	1 600 000	0.5
Germany	5	5 000 000	0.1

The prevalence of T1D among youth in Germany increased similarly from 1.4 per 1000 in 2002 to 2.5 per 1000 in 2020, and although T2D prevalence was considerably lower than in the United States, it again increased more markedly than T1D, from 0.03 to 0.11 per 1000.^5^ Over the period from 1989 to 2013, T1D in youth in Europe increased at an average annual rate of 3.4%, with the annual rate of increase ranging from approximately 1% in Spain and Italy to 6% in Poland, Romania, and Lithuania.^6^

To some extent, the growth in T2D is driven by high rates of obesity. Some 20% of US adolescents aged 10–19 years are obese, with the prevalence increasing from 2000 to 2018 and with obesity rates greater among adolescents from households with lower income or lower parental educational level.^7^ This alone does not seem to fully explain the increase in T2D, however, as it is noteworthy that prediabetes prevalence in adolescents in the United States showed a much greater increase than prevalence of obesity, increasing from 12% in 2000 to 15% in 2005 to 23% in 2011 and to 28% in 2017.^8^ Other factors may need to be considered. Genomic factors may play a role in the risk of T2D development.^9^ The concept of social and ethnic determinants of health has been valuable in understanding disparities in onset of T2D in youth and undoubtedly contributes to the burdens both of T1D and T2D in youth.^10^

Relatively poor glycemic control of youth both with T1D and T2D has been documented in the United States, with mean HbA1c between 8% and 9% and with trends for increasing levels over the past two decades.^11^ The pattern of worsening glycemic control over the past decade among patients with T2D in the United States has also been shown to extend to young adults at ages 20–44.^12^ The difficulty of treatment among youths with T2D is noteworthy. A recent study of insulin and/or metformin‐treated youth with T2D having baseline HbA1c 8%–8.1% showed glycemic worsening with the dipeptidyl peptidase inhibitor linagliptin and only modest improvement with the sodium‐glucose cotransporter‐2 inhibitor empagliflozin.^13^ Along with poor glycemic control there is evidence of development of complications of diabetes in T2D youth. A recent metaanalysis of 27 studies of 5924 youths with T2D showed prevalence of diabetic retinopathy of 2% at <2.5 years diabetes duration, of 5% at 2.5–5 years duration, and of 29% at >5 years duration.^14^ There is evidence of genomic factors contributing to risk of the development of complications in T2D youths.^15^ Understanding this burgeoning epidemic and developing more effective therapeutic approaches will be crucial in the coming years.

## DISCLOSURE

None.
